# Water distribution and association in plant vessels and soil pores in a shrub-encroached grassland

**DOI:** 10.3389/fpls.2025.1595608

**Published:** 2025-07-11

**Authors:** Ruizhe Wang, Jialu Zhang, Yuanming Wang, Xia Hu

**Affiliations:** School of Natural Resources, Faculty of Geographical Science, Beijing Normal University, Beijing, China

**Keywords:** woody plant encroachment, soil pore, soil water, xylem vessel, X-ray computed tomography

## Abstract

Woody plant encroachment is a significant ecological challenge for grassland ecosystems worldwide. Soil water is the major limiting factor for plant growth in arid and semiarid grasslands, which are highly vulnerable to woody plant encroachment. Xylem vessels and soil pores are highly associated between aboveground and belowground systems in relation to water utilization of shrubs. Despite their significant role in water processes, how soil pores and vessels are linked associated with water, is unclear. To address this issue, we quantified structures of soil pores and shrub xylem vessels under different shrub encroachment stages and different soil water conditions (low water, moderate water and field capacity conditions) using the X-ray computed tomography. Results showed that proportions of embolized vessel number peaked in the moderate water state of all water conditions (55.71%). Vessels 10–50 μm in size accounted for over 90% of total vessel numbers, and vessels >20 μm had high water conductivity and were vulnerable to water changes. Irregular pores and pores <30 μm retained water, whereas elongated pores and pores >80 μm were conducive to water movement. Soil porosity was positively correlated with vessel diameter, and the correlation was primarily mediated by root development. Positive correlations occurred between water-filled irregular pores and water-filled vessels, especially those <20 μm. Overall, plants primarily took up water stored in irregular soil pores, and this water was held stably within vessels <20 μm. In the context of climate change, the amplified woody plant encroachment might facilitate the development of xylem vessels and soil porosity, which would accelerate the soil drought.

## 1 Introduction

Woody plant encroachment, featured by the dramatic increase in density, cover and biomass of woody vegetation (van de Koppel et al., 2002; Criado et al., 2020), has become commonplace worldwide especially in arid and semiarid regions (Brandt et al., 2013; Stanton et al., 2018). Woody plant encroachment can significantly alter a variety of ecological processes (Liu et al., 2022), such as microbial habitation (Li et al., 2017), carbon and nitrogen cycling, and soil water retention (Gao et al., 2021; Du et al., 2024). Soil water limitation led to intense water competition among species, which could in turn accelerate the woody plant encroachment (Liu et al., 2021). However, water processes occurring during woody plant encroachment process have not gained sufficient attention (Zhao et al., 2025). Soil pores, shrub root traits and xylem vessels are crucial factors associated with water utilization processes of shrubs. Water transpires from the soil pores into the root, and the subsequent axial water transport is carried out by a network of lignified tubular cells, i.e., vessels (Anadon-Rosell et al., 2018; Hayat et al., 2019). Soil pore characteristics could determine the plant-available water content (Zangiabadi et al., 2020), and vessel anatomical traits mediate water transport and storage (Carvalho et al., 2023; Han et al., 2025). Close hydraulic linkages exist between soil pores and shrub xylem traits, and these linkages might indicate the plant resistance to drought (Liu et al., 2022). Soil microhabitats, which were strongly regulated by pore structure (Bucka et al., 2019), could lead to significant differences in vessel density and mean diameter of shrub species (Ennajeh et al., 2023). The increase in soil porosity could be accompanied by a decrease in vessel diameter (Holste et al., 2006). Overall, exploring the associations between xylem vessels and soil pore structure might contribute to the understanding of how woody plant communities respond to soil water changes during their encroachment, whereas few studies noticed on these crucial associations.

Among angiosperms, water transport occurs primarily through xylem vessels, which connect the stems and roots of plants (Cai and Tyree, 2010; Morris et al., 2018). The size of vessel varies across species and is primarily regulated by the plant size, especially the stem diameter (Olson and Rosell, 2012). Also, high intraspecific variations in vessel traits can occur in response to changes in soil physio-chemical properties, especially soil water content (Ennajeh et al., 2023; Cosmo et al., 2024). Xylem vessel structure is therefore considered a reliable indicator of plant hydraulic properties (Zanne et al., 2010). According to the Hagen–Poiseuille law, the hydraulic conductivity of each xylem vessel is proportional to the fourth power of the vessel diameter (Dodd et al., 2008). Numerous studies have found that soil water limitation could lead to a reduction in vessel diameter and pit membrane area (Kulkarni and Phalke, 2009; Liu et al., 2023). This can be explained that plant growth is regulated by a trade-off between xylem hydraulic conductivity and resistance to embolism (Sperry et al., 1994; Choat et al., 2011). High hydraulic conductivity facilitates efficient water movement and plant growth but may increase vulnerability to cavitation and embolism due to frost and drought (Kirfel et al., 2017). Changes in vessels diameters, connectivity and the thickness of cell walls can be quantified using X-ray computed tomography (CT) (Mayo et al., 2010; Lee et al., 2013; Walker et al., 2022). Previous studies have directly observed that the decreasing soil water content could lead to reduction in vessel cross-section area and increase in vessel density (Thangthong et al., 2019; Garcia-Cervigon et al., 2020). However, the observation of water distribution within vessels remains difficult. This is because of the high overlap between the grayscale values of water and other materials. Overcoming this issue will benefit a more direct analysis on the hydraulic functions of vessels.

Soil pore structure controls many processes in soils, including gaseous exchange, nutrient cycling, and especially water distribution (Rabot et al., 2018). Pore heterogeneity can lead to vertical and horizontal variation in soil moisture (Allire et al., 2009; Wang et al., 2019). For revealing how water moves within soils, attempts have been made to establish the linkages between soil pores and soil water (Tracy et al., 2015). Based on Neutron computed tomography, Mawodza et al. (2020) observed that micropores could be favorable sites for water retention. Based on the single-phase flow simulation, Liu et al. (2024) found that water stored in meso- and macropores were accessible to organisms and plants. Nevertheless, due to the heterogeneous structure of soil (Tracy et al., 2015), it is unclear which types of pores (considering both size and shape) serve as crucial sites for water retention and how they related with xylem vessels. Addressing this issue will facilitate the predictions of water availability of soil, as well as the changes in shrub hydraulic properties during their expansion.

To fill these knowledge gaps, we conducted field sampling of undisturbed soil and plant columns in typical shrub-encroached ecosystems. We also performed CT scanning to quantify the three-dimensional (3D) structure of shrub xylem vessels and soil pores under different soil water conditions. We hypothesized that (i) soil water changes primarily induced changes in number of water-filled vessels >20 μm, (ii) soil micropores and irregular pores favored water retention in that they have highest proportions of water-filled pores in number, and (iii) the abundance of water-filled vessels increased with that of water-filled irregular pores.

## 2 Material and methods

### 2.1 Study site and field sampling

This study was conducted on a typical shrub-encroached grassland in Taibus Banner (115°26′31″E, 42°07′59), Inner Mongolia lies in northern China and has a mean elevation of 1400 m. The region has an arid and semiarid climate, and more than 5.1×10^6^ ha of grassland has been encroached upon by *Caragana microphylla* Lam due to overgrazing, fire management and climate warming (Wigley et al., 2010; Pierce et al., 2018). In this region, previous studies have focused on effects of shrub-encroachment on vegetation diversity (Song and Wang, 2022), soil pore structure and water cycling (Zhou et al., 2019; Zhu et al., 2021). The dominant grass species were *Stipa krylovii*, *Leymus chinensis* and *Artemisia frigida* (Hu et al., 2022). The mean area of and coverage by shrub patches was 9.59 m^2^ and 36.68%, respectively. The typical soil type is chestnut, which is equivalent to Calcic-orthic Aridisol according to the USDA Soil Taxonomy (Soil Survey Staff, 2014).

Three experimental plots representing the degree of woody plant encroachment (from light to severe degree) were selected on the basis of Hu et al. (2015) (**Supplementary Table 1**). Briefly, the three sites (State 1 – State 3) presented similar climatic and pedogenetic conditions but had distinct shrub coverage from 1.32% (State 1), 12.96% (State 2) to 40.12% (State 3). A total of nine undisturbed soil cores (0–50 cm deep) and nine C. *microphylla* samples were collected for each state; 3 replicates of each were used to quantify the soil structure, root traits and shrub xylem vessels via CT scanning. A 300 mm long polyvinyl chloride (PVC) cylinder with an inner diameter of 100 mm was used to collect undisturbed soil samples.

Bulk soils were also collected from each soil layer (10 cm intervals) for measurement of basic soil physiochemical properties. In the laboratory, bulk soil samples from the same locations were mixed and sieved through a 2-mm mesh to remove stones, plant residues, and roots (Wang and Hu, 2024). The sieve-pipette method was used to determine the particle size distribution (Mako et al., 2019). The soil bulk density was measured with Cylinder method (Wang and Hu, 2023). Soil pH measurement was conducted by an FE20 pH meter (Mettler Toledo, Columbus, USA) from slurries of samples in a soil:water ratio of 1:2.5 (w:w) (Huang et al., 2024). Soil organic carbon (SOC) content was determined using the CN802 elemental analyzer (VELP, Italy). Basic soil properties are shown in **Table 1**.

**Table 1:** Basic soil physio-chemical properties of different sites.

Soil depth (cm)	Bulk density (g/cm^3^)	Soil organic carbon content (g/kg)	pH	Particle size composition (%)		
				Clay	Silt	Sand
0-10	1.42 ± 0.11	19.55 ± 2.75	8.27 ± 0.11	2.98 ± 0.60	10.94 ± 2.91	86.07 ± 3.50
10-20	1.52 ± 0.10	16.23 ± 3.24	8.37 ± 0.23	2.80 ± 0.23	10.10 ± 1.71	87.10 ± 1.94
20-30	1.46 ± 0.12	19.13 ± 4.44	8.34 ± 0.12	3.45 ± 0.49	11.06 ± 2.65	85.48 ± 3.14

All data is presented with standard error (n=3).

**Figure 1:**
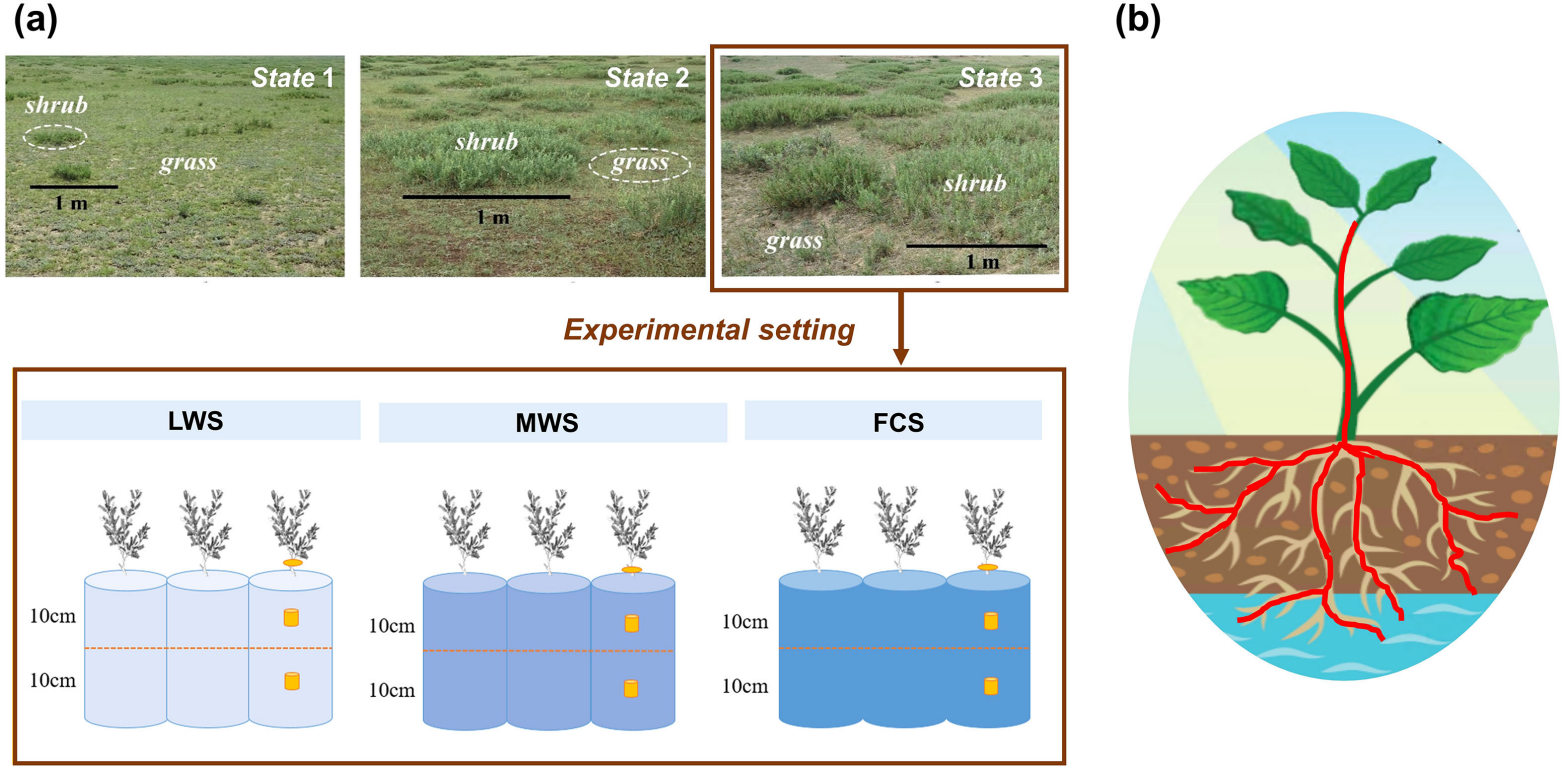
**(a)** Landscape of sampling sites of shrub encroachment State 1, State 2 and State 3 and experimental design for preparing the columns for CT scanning and **(b)** conceptual diagram of the connection between aboveground plants and underground soil. LWS, low water state; MWS, moderate water state and FCS, field capacity state. Red represents water.

Additionally, nine soil columns for C. *microphylla* in State 3 were excavated to quantify the water distribution in the xylem vessels and water-filled pores using CT (**Figure 1a**). Three typical shrub-encroached plots (50×50 m) were selected, and three C. *microphylla* plants with similar growth statuses were selected at intervals of 5–10 m in each study plot; below these plots, intact soil cores with C. *microphylla* were also collected. The cores were obtained with a PVC cylinder (100 mm in external diameter and 300 mm in length) with a beveled edge at its bottom. Core extraction was performed according Sammartino et al. (2012). The cores were preserved in an icebox at 4°C for transport to the laboratory.

### 2.2 Experimental design

In the laboratory, the nine columns were labeled and divided into three groups for setting distinct soil water conditions (**Figure 1**). The soil water content for the first group of columns (no. 1–no. 3) was measured. This group was subsequently maintained under natural water conditions and represented a low water state (LWS). A second group of columns (no. 4–no. 6) were set to the field capacity state (FCS) through the gravity drainage method, and this group represented a high water condition. After the difference in column weights between the FCS and LWS was calculated, the drip irrigation method was used to control the weight of the columns. A third group of columns (no. 7–no. 9) were set to a moderate water state (MWS).

For every column, undisturbed soil cores from the 0–10 cm and 10–20 cm soil layers were collected with PVC pipes (2.5 cm in height and 2 cm in inner diameter). A total of 18 soil cores were obtained (3 groups × 2 layers × 3 replicates) and were prepared for quantification of water-filled soil pores via CT scanning. Additionally, the aboveground parts of the plants were removed, and a 3 cm section from the middle of each stem was cut underwater. To avoid water loss, the ends of the stem were sealed with Parafilm sealing film before they were scanned by the CT to quantify the water-filled xylem vessels (Savi et al., 2017).

### 2.3 CT scanning and image processing

#### 2.3.1 Quantification of soil pore structure and root parameters

CT scanning was performed on soil columns collected in the field, soil cores from different water treatments, and xylem vessels. The soil columns were scanned using a GeoScan-2000 X-ray microscope (Sanying Precision Instruments Co., Ltd., China) at an energy level of 175 kV and 450 μA to analyze the soil pore structure. After scanning, 1920 × 1536-pixel images were obtained. After the images were reconstructed, 1800 × 1800 images with a slice thickness of 0.066 mm were obtained. The spatial resolution of the images was 66 μm.

We performed image visualization and data analysis with Avizo 9.0 (FEI). The method used by Luo et al. (2010) was followed in the interpretation of the soil pores. A cylindrical cropping tool was used to obtain a region of interest (ROI), and the area outside the ROI was deleted to exclude the voids near the column walls and to remove the sampling disturbances. We fitted a parabola according to the intensity histogram of the entire soil column to extract pores and adjusted it according to the intensity histogram of each soil column (Katuwal et al., 2015). The selected threshold value was subsequently applied to all slices of the ROI to binarize the data. The 3D soil pore networks were visualized using the “Volume Rendering” tool. The skeletonization of pores was necessary to accurately quantify the topology and length and to visualize the networks in three dimensions. The “Label Analysis” and “Auto Skeleton” tools were used to calculate pore parameters (e.g. equivalent diameter, number density, surface area density, and node density) (Hu et al., 2023; Luo et al., 2010; Yang et al., 2021). The soil pore characteristics can be seen in **Supplementary Table 2**.

The number density (ND,×10^5^ no.m^-3^) of pores was calculated as following Equation 1:


(1) $$\begin{array}{c} ND=\frac{N_{m}}{V} \end{array}$$

where $N_{m}$ (−) is the number of pores, and *V* (m^-3^) is the volume of the ROI.

One pore network may consist of several branches of connected pores or just one individual pore. The branch density (BD, no.m^-3^) is the number of branches in a unit volume, which can be calculated as following Equation 2:


(2) $$\begin{array}{c} BD=\frac{n}{V} \end{array}$$

where n (−) is the total pore number in the i th branch, and *V* (m^-3^) is the volume of the ROI.

The surface area density (SD, m^2^ m^-3^) was calculated as following Equation 3:


(3) $$\begin{array}{c} SD=\frac{S_{m}}{V} \end{array}$$

where $\;S_{m}$ (m^2^) is the total pore surface area in the *i*th branch, and *V* (m^-3^) is the ROI volume.

The node density (NoD, no.m^-3^) is the number of nodes in a unit volume, which can be calculated as following Equation 4:


(4) $$\begin{array}{c} NoD=\frac{N_{n}}{V} \end{array}$$

where $N_{n}$ (−) is the number of nodes, and V (m^-3^) is the volume of the ROI.

The inclination of a pore branch was characterized by an angle away from the vertical direction. The mean angle (MA, ° )was calculated as following Equation 5:


(5) $$\begin{array}{c} MA=\frac{\sum_{i=1}^{n}L_{ti}\theta_{i}}{\sum_{i=1}^{n}L_{li}} \end{array}$$

where *i* is the index of a macropore in the i th branch, $L_{t}$ (mm) is the total actual pore length in the i th branch, $L_{i}$ (mm) is the total straight-line distance of all the pores in the i th branch, and $\theta_{i}\;({}^{\circ})$ is the angle in the i th branch.

The mean pore coordination number (CN, no. mm^-3^) was calculated as following Equation 6:


(6) $$\begin{array}{c} CN=\frac{\rho_{b}}{N_{n}} \end{array}$$

where $\;\rho_{b}$ (no. mm^-3^) is the branch density, and $\;N_{n}$ (−) is the number of nodes.

The hydraulic radius (HR, mm) was calculated as following Equation 7:


(7) $$\begin{array}{c} HR=\sqrt{\frac{V_{t}}{L_{t}\pi }} \end{array}$$

where $V_{t}$ (mm^-3^) is the total volume, and $L_{t}$ (mm) is the total actual pore length in the i th branch.

Root is the primary organ through which shrubs directly obtain water from soil pores (Chen et al., 2024). Root and stem traits are functionally coordinated to maximize the efficiency of acquiring and using limited sources (Kramer-Walter et al., 2016). We therefore quantified the 3D root characteristics within soil columns via CT scanning. Briefly, roots were interpreted mainly by the growth method (Kuka et al., 2013). After selecting the threshold, the magic wand tool was used to select the roots in images before they were enlarged to ensure that the valid inner voxels were counted as roots. Consequently, root structural parameters, including root volume density, root surface area density, and root mean radius, etc., were calculated.

#### 2.3.2 Quantification of water-filled soil pores

The soil cores obtained after the experiments were scanned using a nanoVoxel-2000 X-ray 3D microscope (Sanying Precision Instrument Co., Ltd., China) at an energy level of 150 kV and 50 μA to determine the water distribution in the soil pores. A 5-cm segment of the stem, located near the base and corresponding to the soil column, was cut using underwater cutting. The water distribution of vessels was immediately scanned using a nanoVoxel-3000 X-ray 3D microscope (Sanying Precision Instrument Co., Ltd., China) with an energy level of 90 kV and 50 μA. The resolution of the small soil cores was 6 μm.

**Figure 2:**
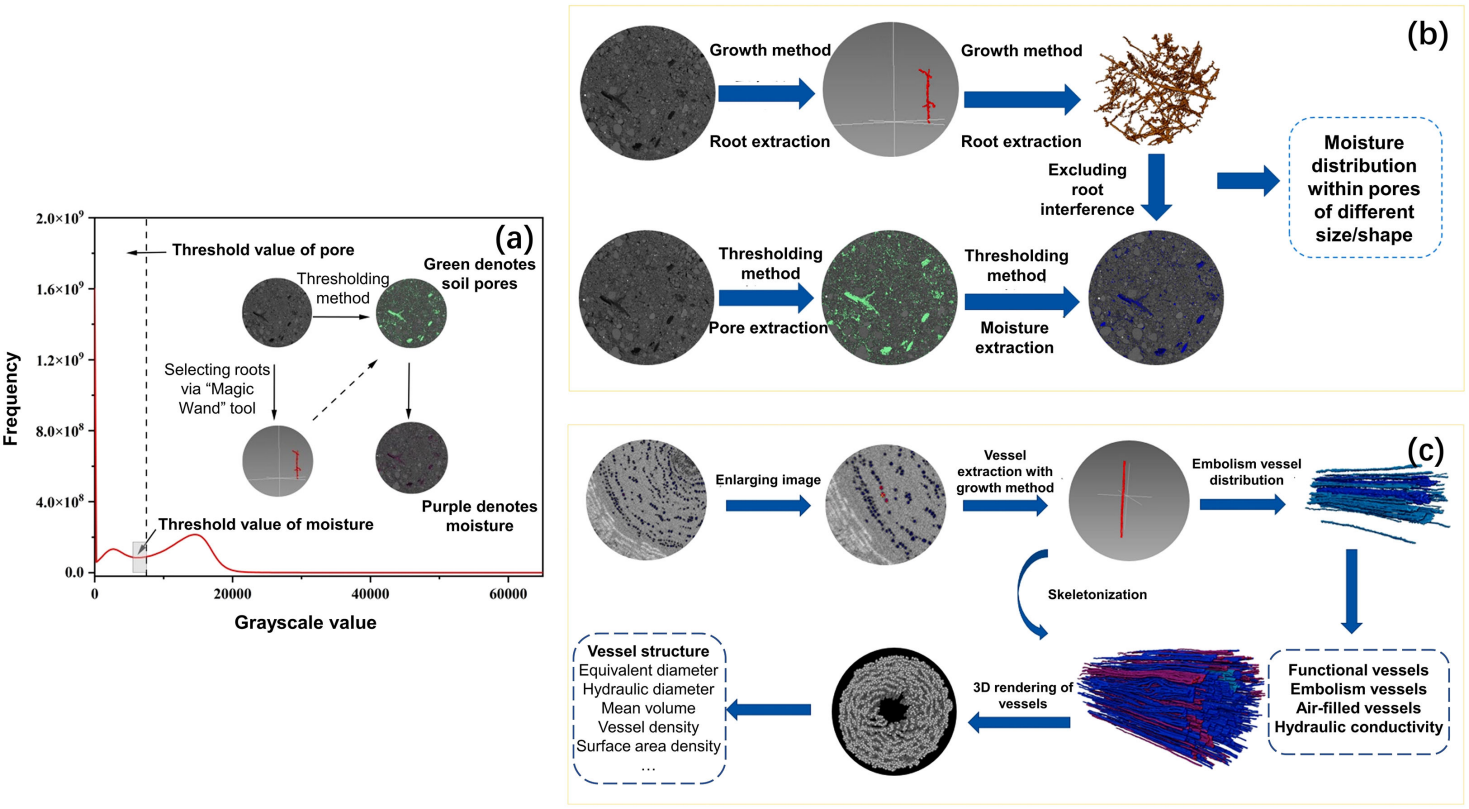
Thresholding value for soil pores and water **(a)**, procedures used for the visualization and quantification of soil water distribution **(b)** and xylem vessels **(c)**.

The soil pores and water content were interpreted in the similar way as the soil pores; specifically, to crop and acquire the ROI, appropriate threshold based on the intensity histogram was chosen to extract the pores and water (**Figures 2a, b**). However, because they partially overlapped with the threshold range of the root system, we excluded the impact of root system on the data. We used the global threshold method to identify soil pores and moisture levels. After the growth and threshold approaches were combined, the shrub roots were measured (Hu et al., 2019). Then, we improved the accuracy of the threshold method by eliminating the intersection between the pores and the root system via the “and not image” function. Consequently, the indicators of soil pores and water were calculated via the “Label Analysis”.

To investigate changes in water distribution associated with the pore network, the pores were divided into different groups, and their volume, number, and the proportion of water-filled pores (PWP) were calculated. Pores were classified into three categories according to diameter: macropores (>80 μm), mesopores (30–80 μm) and micropores (5–30 μm) (Wang and Hu, 2023). Pores were also divided into three groups on the basis of their pore shape factor (SF): irregular pores (SF < 0.5), near-spherical pores (0.5 < SF < 1) and elongated pores (SF > 1) (Wu et al., 2022). The SF can be calculated as following Equation 8:


(8) $$\begin{array}{c} SF=\frac{S_{p}^{3}}{36\pi \times V_{p}^{2}} \end{array}$$

where $S_{p}$ (mm^2^) and $\;V_{p}$ (mm^3^) represent the surface area and volume of pores, respectively.

The PWP was calculated as following Equation 9:


(9) $$\begin{array}{c} PWP=\frac{V_{w}}{V_{p}} \end{array}$$

Where $V_{w}\;$ and $V_{p}\;$ represent the volume of water and pores, respectively.

#### 2.3.3 Quantification of shrub xylem vessels

Shrub stems were scanned using a nanoVoxel-3000 X-ray microscope (Sanying Precision Instruments Co., Ltd., China), and the middle of the plant stem segments was scanned at an energy level of 25 kV and 200 μA to obtain the xylem vessel structure. To prevent interference from water loss during the scanning process and to improve the contrast of the scanned image, the samples were dried at 25°C for 6 hours. A total of 2940 × 2304 pixel images were produced. Then, 2500 × 2500 images with a slice thickness of 0.003 mm were produced after reconstruction. The voxel size was 0.003 mm × 0.003 mm × 0.003 mm, with the spatial resolution of 3 μm.

Xylem vessels include embolized vessels and functional (water-filled) vessels. Owing to the overlap of the grayscale value range of water-filled vessels and other components of vessels, we extracted embolized vessels and completely dehydrated vessels to indicate the structure and water distribution of vessels (**Figure 2C**). The growth method was used for segmentation and extraction because vessels extended along the slices. We used the magic wand tool in Avizo 9.0 to select voxel points for each vessel and repeated expansion and erosion until the complete vessel was displayed in the window. 3D volume rendering and segmentation of the embolized vessels were subsequently performed after binarization. To accurately quantify the 3D structural data of the xylem vessels, they were skeletonized. To obtain full embolized vessels, the stem segments were dried at 25°C for 24 hours. Using the “and not image” function, the overlapped part of all embolized vessels and the embolized vessels in the natural state of plants were excluded to obtain the water-filled vessels.

Ultimately, we obtained 2D vessel parameters, including equivalent vessel diameters and hydraulic diameters, as well as important 3D vessel network data (equivalent circle diameter, hydraulic diameter, vessel diameter contributing to 95% of the hydraulic conductance, mean vessel area, mean vessel number, vessel density, and vessel lumen fraction). The consistency between the X-ray micrograph images and cross-sectional images was verified (Skelton et al., 2017).

For vessel parameters, the equivalent circle diameter is the equivalent area circle diameter of the vessel. The mean vessel area and mean vessel number are the average area and average number of vessels in the stem cross-section, respectively. Vessel density was defined as the number of vessels per unit stem cross-section. The vessel lumen fraction is the ratio of the total vessel lumen area in the stem cross-section to the stem cross-sectional area. The hydraulic diameter (D_h_, μm), which reflects the hydraulic conductivity of vessels, was calculated as following Equation 10:


(10) $$\begin{array}{c} {\text{D}}_{h}=\sqrt[{4}]{\frac{\sum_{i=1}^{n}D^{4}}{n}} \end{array}$$

Where *n* is the total vessel number, *D* (μm) represents the equivalent diameter.

The vessel diameter that contributed to 95% of the hydraulic conductance (D_95%_) was calculated by sorting the vessel diameters measured in the stem cross-section from largest to smallest, calculating each equivalent circle diameter by the fourth power ( ${\text{D}}^{4}$ ), and dividing them from smallest to largest ( $\sum {\text{D}}^{4}$ ) until it equalled 95% of the total $\sum_{i=1}^{\text{n}}{\text{D}}^{4}$ ; finally, the average vessel diameter was calculated as D_95%_. This parameter also reflects the ability of a vessel to transport water.

The 2D vessel parameters were used to estimate the total hydraulic conductivity (K, kg s^-1^m^-1^Mpa^-1^) of each cross-section using the Poiseuille equation (Equation 11):


(11) $$\begin{array}{c} K=\sum_{i=1}^{n}\frac{\pi d_{i}^{4}}{128\eta } \end{array}$$

Where *d* represents the equivalent diameter of vessels, and η represents the viscosity of water at 20 °C (1.002×10–^9^ Mpa^-1^).

The maximum hydraulic conductivity (*K_m_*) of each vessel was quantified through images of fully embolism vessels. The natural hydraulic conductivity (*K_n_*) was obtained by subtracting the theoretical hydraulic conductivity (*K_s_*) of the embolized vessels corresponding to each stem under different soil water conditions from the maximum hydraulic conductivity.

### 2.4 Statistical analysis

IBM SPSS 20 (SPSS Inc., USA) was used for all the statistical analysis. All experimental data were assessed for a normal distribution via a Shapiro–Wilk normality test. One–way ANOVA followed by Fisher’s protected least significant difference (LSD) test was performed to examine whether the differences in hydraulic conductivity, water-filled pores and functional vessels under different water conditions were significant at the level of *p*<0.05. The relationships between vessel structure, pore characteristics and root characteristics were examined via Pearson’s correlation analysis. Univariate regression was performed to quantify the relationships between the functional vessels and water-filled soil pores.

## 3 Results

### 3.1 Xylem vessel and root structure of woody plants

**Figure 3:**
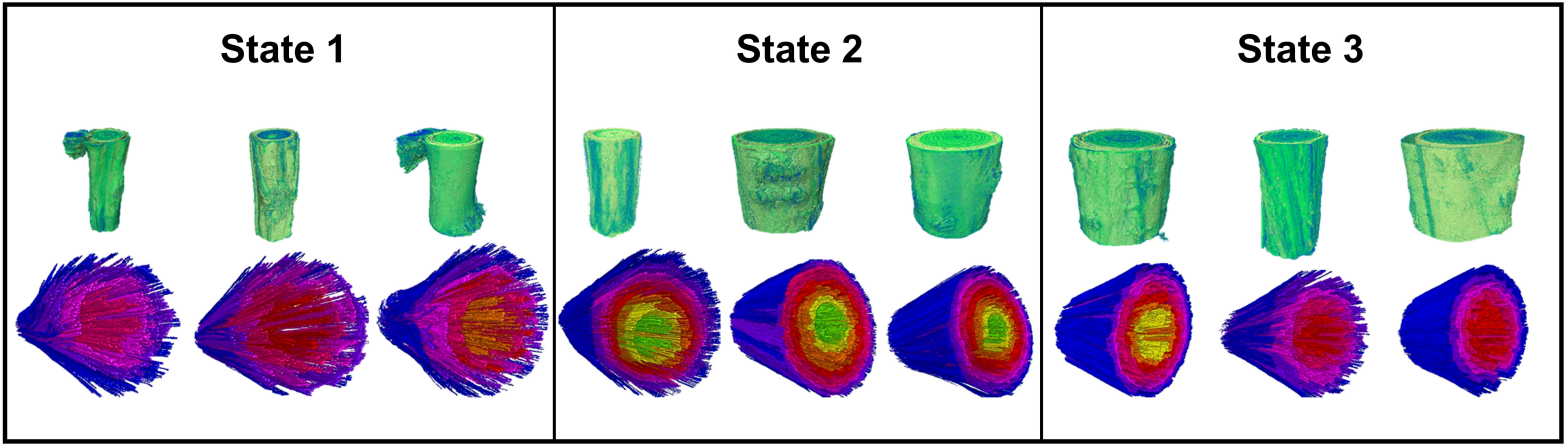
3D visualization of xylem vessels from the shrub encroachment State 1, 2 and 3 (the adjacent vessels in space are rendered in the same color).

**Figure 3** shows the visualization of the total xylem vessel networks of C. *microphylla* stems of three shrub encroachment degrees. Vessels spread from the center to the edges with no obvious patterns in spatial distribution. Nearby vessels were interconnected to form clusters. The vessels of shrubs from State 3 had significantly greater equivalent diameters (25.38 μm), hydraulic diameters (31.70 μm), mean vessel area (599.01 μm^2^) and mean lumen fractions (0.09 μm^2^/μm^2^) compared to those from State 1 and 2 (*p*<0.05) (**Table 2**). These results indicated the xylem vessel size increased from State 1 to State 3. For 3D root parameters, woody plant encroachment mainly impacted the root branch density (**Table 3**). The root branch density of shrubs decreased significantly from State 1 (703.33×10^5^ no./m^3^) to State 3 (68.85×10^5^ no./m^3^) (*p*<0.05).

**Table 2:** Xylem vessel characteristics of *C. microphylla* in different shrub-encroachment states.

Shrub encroachment states	Equivalent diameter (μm)	Hydraulic diameter (μm)	D_95%_ (μm)	Mean vessel area (μm^2^)	Mean vessel number (no)	Vessel density (no. mm^-2^)	Vessel lumen fraction (μm^2^ μm^-2^)
State 1	16.88 ± 0.23b	20.90 ± 0.86b	22.00 ± 1.03	263.39 ± 13.06b	1070 ± 103	134.33 ± 37.19	0.04 ± 0.01b
State 2	19.37 ± 2.04b	23.19 ± 1.70b	24.28 ± 1.21	339.86 ± 60.80b	3462 ± 1219	169.67 ± 46.94	0.06 ± 0.01ab
State 3	25.38 ± 2.56a	31.70 ± 3.60a	34.57 ± 4.04	599.01 ± 114.75a	4167 ± 1877	144.67 ± 10.21	0.09 ± 0.02a

The data represent the mean ± standard deviation (n=3). Different letters in the same column indicate significant differences in the parameters between states at the level of p<0.05.

**Table 3:** The 3D root structural characteristics in different shrub-encroachment states.

State	Volume density (mm^3^/mm^3^)	Surface area density (mm^2^/mm^3^)	Branch density (×10^5^ no./m^3^)	Node density (×10^5^ no./m^3^)	Mean angle (°)	Mean radius (mm)
State 1	0.0019 ± 0.0004	0.022 ± 0.003	703.33 ± 392.33a	139.90 ± 42.20	49.72 ± 8.53	0.05 ± 0.03
State 2	0.0029 ± 0.0010	0.020 ± 0.002	376.56 ± 194.95ab	133.75 ± 60.91	60.40 ± 3.50	0.13 ± 0.06
State 3	0.0036 ± 0.0018	0.015 ± 0.009	68.85 ± 53.00b	31.42 ± 22.44	40.43 ± 25.56	0.26 ± 0.18

All data was presented with standard error (n=3). Different lowercase note represents significant differences in parameters of state 1, 2 and 3 (*p*<0.05).

**Figure 4:**
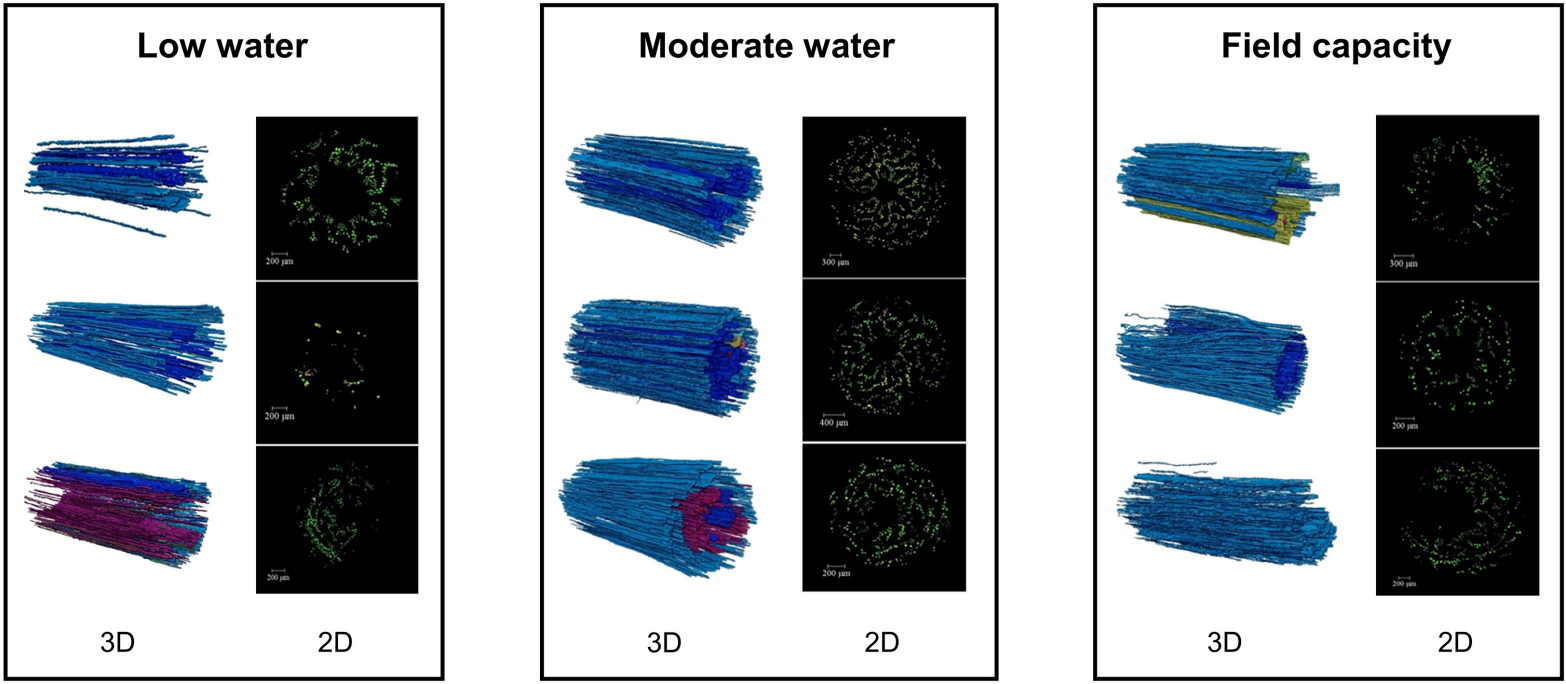
The 2D and 3D visualization of embolized xylem vessels with different soil water conditions treatments (the adjacent vessels in space are rendered in the same color).

The visualization of embolized vessels under different soil water conditions is shown in **Figure 4**. The low water state presented few embolized vessels (24.00%, **Table 4**) and most of them concentrated in the middle part of xylems. In the moderate water and field capacity states, embolism spread from the middle to the exterior part within vessels. The proportions of embolized vessels followed: MWS (55.71%) > FCS (34.27%) > LWS (24.00%) (**Table 4**). The vessel structural parameters, including the equivalent diameter, hydraulic diameter, volume density, and length density, did not change significantly as the water content increased (**Table 4**). Changes in soil water content did not impact the hydraulic conductivity of vessels (**Table 4**). Overall, the structure and function of xylem vessels remained relatively stable as water levels fluctuated. Of three soil water conditions, embolism was most noticeable in the moderate water state.

**Table 4:** Structural characteristics and hydraulic conductivity of xylem vessels under different soil water conditions.

Water condition	Equivalent diameter (μm)	Hydraulic diameter (μm)	Volume density (μm^3^/cm^3^)	Cross-section area (×10^4^ μm^2^)	Length density (×10^8^μm/cm^3^)	Surface area density (×10^9^ µm^2^·cm^-3^)	branch density (no·cm^-3^)	Embolism rate (%)	Natural hydraulic conductivity (K_n_, kg×s^-1^×m^-1^×MPa^-1^)	Theoretical hydraulic conductivity (K_t_, kg×s^-1^×m^-1^×MPa^-1^)	Water-filled vessel number (no.)
LWS	24.17 ± 3.08	39.12 ± 7.14	1165.968 ± 799.612	1.04 ± 0.34	5.38 ± 4.07	5.537 ± 3.744	392.683 ± 197.498	24.00 ± 10.37b	0.64 ± 0.32	0.89 ± 0.25	1227.22 ± 221.52
MWS	29.80 ± 8.16	38.36 ± 7.68	1176.054 ± 732.275	1.59 ± 0.45	6.16 ± 5.92	7.255 ± 2.552	833.141 ± 13.168	55.71 ± 14.23a	0.70 ± 0.32	1.77 ± 0.98	976.00 ± 19.06
FCS	23.87 ± 0.35	33.49 ± 9.52	1141.547 ± 109.257	1.29 ± 0.09	3.37 ± 1.61	4.784 ± 4.769	617.770 ± 48.602	34.27 ± 4.67b	0.49 ± 0.21	0.92 ± 0.32	1213.22 ± 154.68

All data was presented with standard error (n=3). Different lowercase letters denote significant differences in vessel characteristics of different water conditions.

### 3.2 Water distribution of xylem vessels

**Figure 5:**
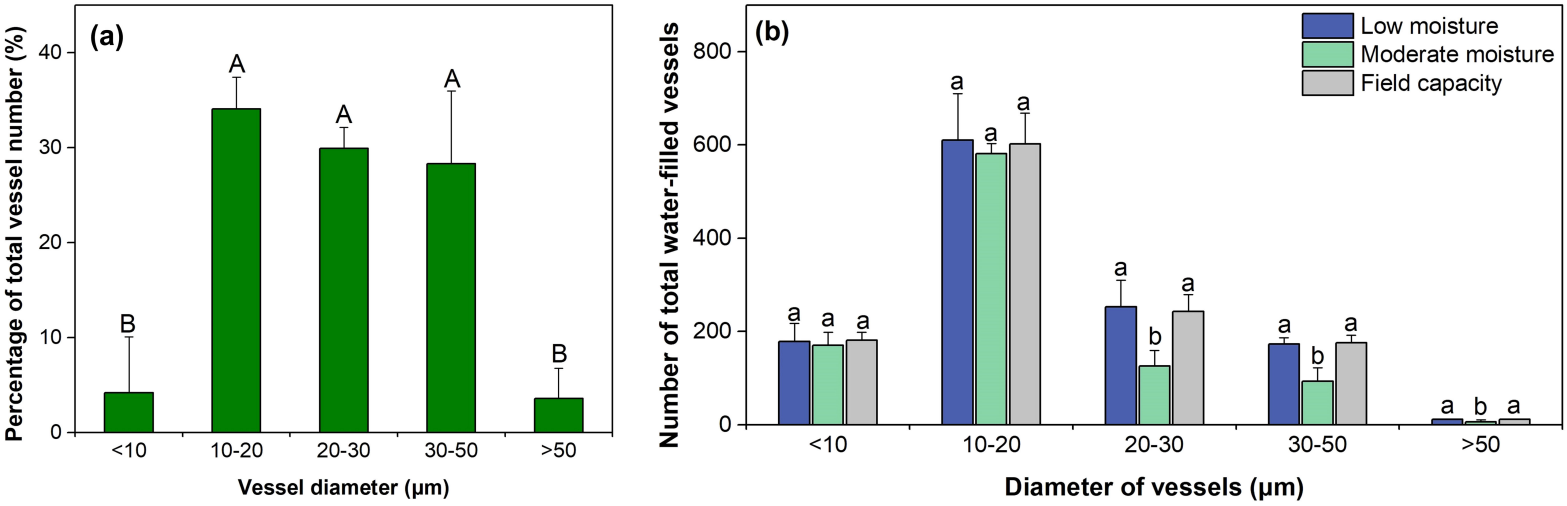
Distribution of vessels with different diameters **(a)** and the number of functional vessels of different diameters **(b)** in the xylem. Different uppercase letters in **(a)** denote significant differences among diameter classes (*p*< 0.05). Different lowercase letters in **(b)** denote significant differences among water conditions (*p*< 0.05). The bars represent the means ± standard errors (n=3).

In *C. microphylla* stems, the diameters of most vessels were between 10–50 μm, accounting for 92.26% of the total number of vessels on average (**Figure 5a**). Vessels with a diameter of 10–20 µm had the greatest number of all water-filled vessels under all water conditions (610.11, 581.33, and 602.22 no. for the LWS, MWS and FCS, respectively) (**Figure 5b**). For vessels > 20 μm, the number of water-filled vessels in the MWS (126.11, 92.89 and 5.78 for vessels 20–30 μm, 30–50 μm and >50 μm, respectively) was significantly lower than those in the LWS and FCS (*p*<0.05). The number of <20 μm water-filled vessels did not change significantly with water conditions, which indicates that vessels of this diameter had a stable water-holding capacity.

### 3.3 Soil pore structure and water distribution

**Figure 6:**
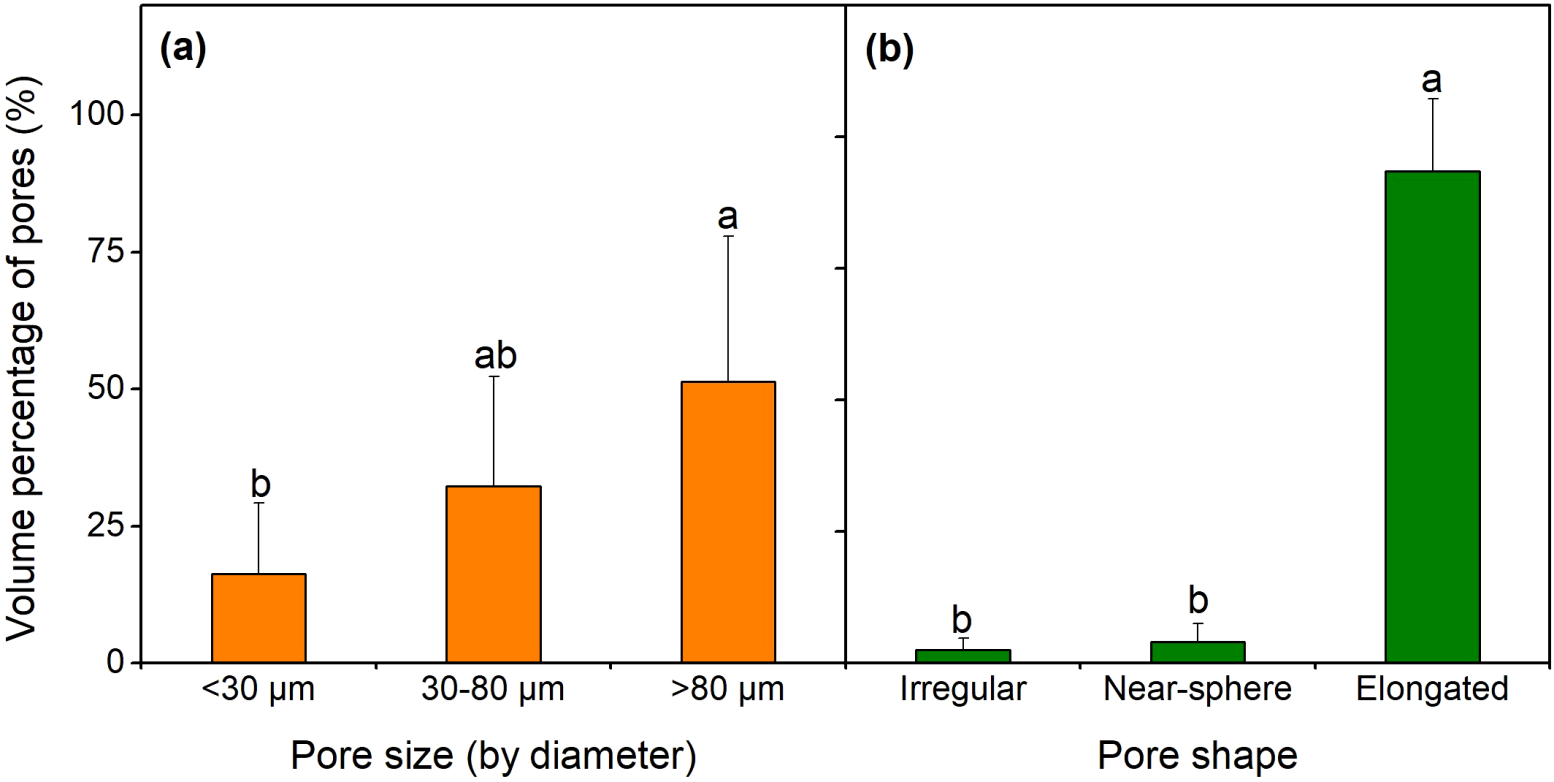
Pore size distribution **(a)** and pore shape distribution **(b)** in the soil. The bars represent the means ± standard errors (n = 3). Different lowercase letters denote significant differences among groups (*p* < 0.05).

**Figure 7:**
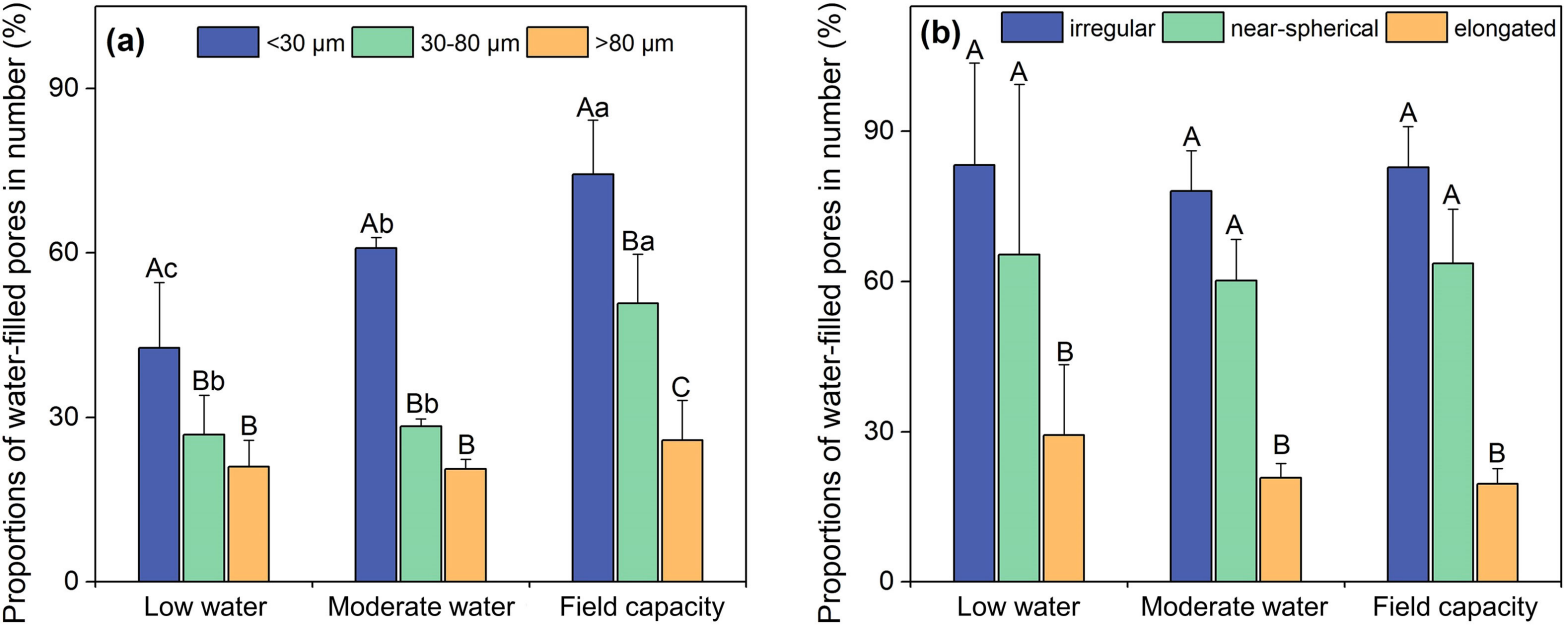
Proportions of water-filled pores in number with different **(a)** pore diameters and **(b)** pore shapes. Different uppercase letters denote significant differences among pore size classes (or pore shape classes) (*p* < 0.05). Different lowercase letters denote significant differences among water conditions (*p* < 0.05).

Over 50% of the soil pore space was occupied by pores > 80 μm, followed by pores 30–80 and < 30 μm, with the porosity of 0.924×10^-3^ mm^3^/mm^3^ and 1.466×10^-3^ mm^3^/mm^3^, respectively (**Figure 6a**). Among all the pore shapes, elongated pores had the largest porosity (2.671×10^-3^ mm^3^/mm^3^, **Figure 6b**), occupying over 90% of the total pore space. The proportions of water-filled pores (by number) varied dramatically according to pore shape, pore size, and soil water conditions (**Figure 7**). Among all the pore sizes, pores <30 μm had the greatest proportion of water-filled pores, which increased with soil water content from 42.65% in the LWS to 74.34% in the FCS. Pores > 80 μm had the lowest water-filled pore proportion (22.50% on average), significantly lower than others (*p*<0.05). Proportions of water-filled irregular, near-spherical and elongated pores were not altered by soil water content, with the proportions as follows: irregular pores (80.38%) > near spherical pores (63.09%) > elongated pores (23.24%). These results indicate that micropores and irregular pores might favor water storage.

### 3.4 Interactions between vessels, soil pores and roots

**Table 5** shows the correlations between vessel structural parameters and soil pore characteristics. Vessel equivalent diameter, D_95%_, and surface area density were significantly and positively correlated with soil porosity (*p*<0.05). Negative associations were observed between vessel equivalent diameter, hydraulic diameter, D_95%_, surface area density, and pore coordination number (*p*<0.05). No obvious correlations were observed between vessel hydraulic conductivity and pore parameters. The correlations between root parameters and vessel characteristics is shown in **Table 6**. Vessel surface area, number density and vessel density were positively correlated with root volume density (*p*<0.05).

**Table 5:** Correlations between soil pore characteristics and xylem vessel characteristics.

Pore parameter	Vessel parameter					
	Equivalent diameter	Hydraulic diameter	D_95%_	Surface area	Number density	Vessel density
Porosity	0.699*	0.661	0.672*	0.702*	0.340	0.023
Surface area density	0.525	0.481	0.496	0.529	0.144	0.023
Number density	0.546	0.517	0.539	0.554	0.123	−0.060
Branch density	0.335	0.274	0.284	0.333	0.032	−0.077
Length density	0.655	0.585	0.571	0.638	0.472	−0.098
Node density	0.456	0.410	0.426	0.460	0.074	−0.070
Hydraulic radius	0.571	0.555	0.577	0.581	0.248	0.284
Tortuosity	−0.634	−0.636	−0.655	−0.648	−0.227	0.038
Connectivity	−0.166	−0.197	−0.175	−0.164	−0.059	0.471
Equivalent diameter	0.661	0.656	0.640	0.656	0.358	0.049
Mean volume	−0.294	−0.313	−0.330	−0.313	0.037	0.364
Mean angle	−0.139	−0.138	−0.119	−0.142	0.078	−0.229
Coordination number	−0.745*	**−0.742**	−0.752*	−0.753*	−0.385	−0.025

The bold values with * denote that the correlation is significant at the level of *p*<0.05 (n=9).

**Table 6:** Correlations between root characteristics and xylem vessel characteristics.

Root parameter	Vessel parameter					
	Equivalent diameter	Hydraulic diameter	D_95%_	Surface area	Number density	Vessel density
Volume density	0.450	-0.400	-0.450	0.667*	0.750*	0.717*
Surface area density	0.200	-0.033	-0.067	0.617	0.433	0.667*
Branch density	-0.167	0.067	-0.100	0.450	0.283	0.083
Node density	-0.533	-0.333	-0.500	0.367	0.350	0.417
Mean angle	0.117	0.267	0.433	-0.333	0.750*	0.717*
Mean radius	-0.217	-0.133	-0.583	-0.217	0.133	-0.233

The bold values with * denote that the correlation is significant at the level of *p*<0.05 (n=9).

**Figure 8:**
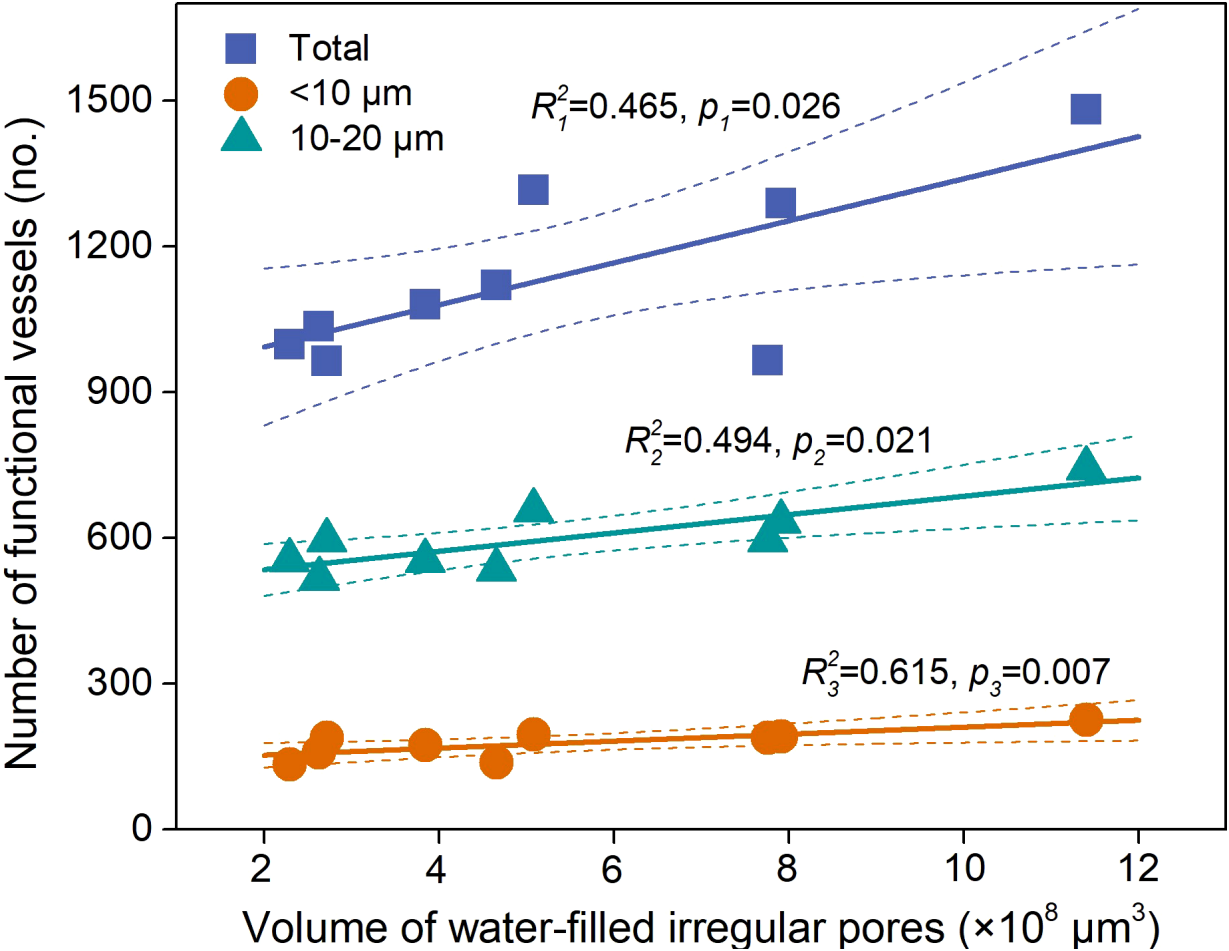
Relationships between water-filled irregular pores and water-filled vessels (n=9). The dashed line represents the 95% confidence interval of the linear fit.

We tried to establish connections between water-filled soil pores and water-filled xylem vessels. A significant positive relationship was observed between total water-filled vessels and irregular pore volume (**Figure 7**; *p*=0.026). Specifically, irregular pore volume had a positive effect on <10 μm (*p*=0.007) and 10–20 μm (*p*=0.021) water-filled vessels (**Figure 8**). Therefore, irregular pores may be the dominant site for water uptake by stems.

## 4 Discussion

Findings of the study verified our hypotheses. Soil water changes primarily altered number of water-filled vessels >20 μm. Previous studies have shown that an increase in vessel diameter is accompanied by decreasing drought resilience and water use efficiency in plants (Lobo et al., 2018; Garcia-Cervigon et al., 2020; Levionnois et al., 2021). This can be explained that large vessels containing more vessel wall area and more inter-vessel pits and therefore predicted to be more likely to contain a large and vulnerable pit membrane pore (Wheeler et al., 2005; Christian et al., 2012). According to Young-Laplace equation, narrow vessels favor water storage via stronger capillary forces due to higher contact area with moisture (Mrad et al., 2018). Hence, vessels <20 μm had lower water conductivity efficiency but greater water storage compared with larger vessels, making them more resistant to embolism (Lens et al., 2022).

Consistent with our second hypothesis, pores <30 μm and irregular pores had the highest proportions of water-filled pores. Previous studies have confirmed that larger pores (>50 μm) are essential for infiltration, drainage and aeration, whereas micropores are crucial for water storage as they could store water at relative high suctions and form a water film (Hernandez et al., 2019; Mawodza et al., 2020; Kim et al., 2021). The proportion of water-filled pores increased dramatically with increasing soil water content, and when water conditions change, micropores and macropores may act as primary sites for water accumulation and water loss, respectively (Lu et al., 2017). Among all pore shapes, irregular pores had a relatively high water retention capacity. Mawodza et al. (2020) reported that water was largely localized in regions with abundant near-spherical and irregular pores, which mainly existed within soil aggregates and hindered water movement. Elongated pores had the lowest water-filled pore volume. In shrub-encroached grasslands, elongated pores (especially with diameters >80 μm) were primarily exerted by root development, and these pores serve as the main pathway for preferential flow in deep soil layers (Hu et al., 2022). Overall, water retention capacity of pores is highly dependent on their size and morphology.

**Figure 9:**
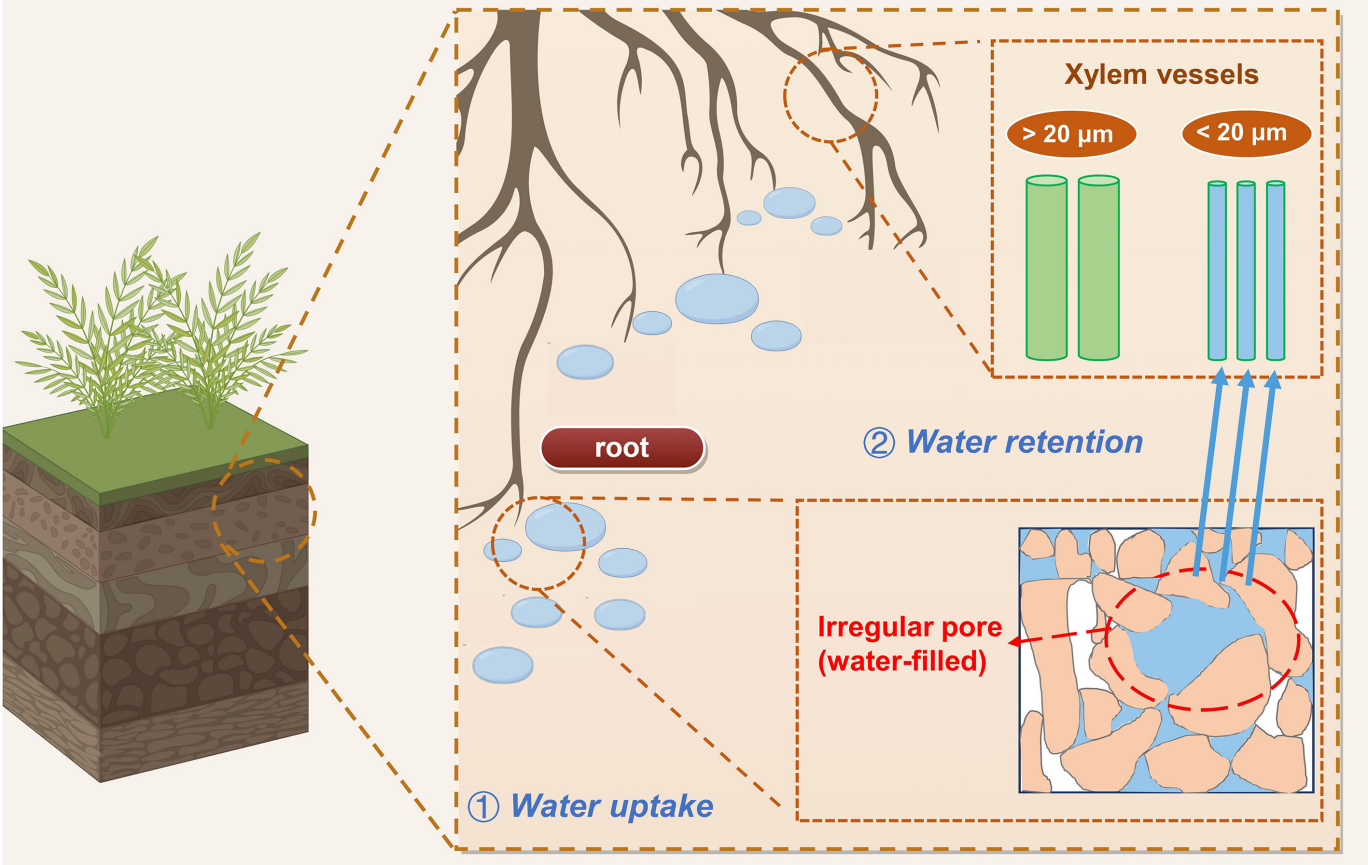
Mechanistic diagram of the connections between plant xylem vessels and soil pores associated with water. Blue denotes water.

Relationships between xylem vessels and soil pores can be established via root penetration and water uptake of shrub. Our results revealed positive correlations between soil porosity and vessel diameter, was primarily mediated by roots. In shrubland ecosystems, root growth is the key regulator on the increase in soil porosity (Hu et al., 2019). In our study, vessel surface area, number density and vessel density were positively correlated with root volume density (*p*<0.05, **Table 5**), similar to the findings of Kirfel et al. (2017) that vessel size could be a function of root size. Nisa et al. (2024) also reported the positive correlation between root size and vessel size. Arid and semi-arid environments could exert stronger coordination of stems and roots for better acquisition of water (Shen et al., 2019). Greater root volume reflected a large root system that could explore greater water supply to stems, and thus promotes the increase in vessel size (Filho et al., 2021). Therefore, increase in soil porosity is accompanied by the intensified root development, and thus could lead to expansion in root size to improve available water use efficiency (Omondi et al., 2016; Day et al., 2023). Also, consistent with our third hypothesis, significant positive relationships existed between water-filled irregular pores and water-filled vessels (especially vessels <20 μm). Hence, irregular pores can be crucial indicators of the amount of available water for plants. We propose a mechanism of vessel–pore interactions associated with water: plant roots take up water stored in irregular soil pores, and subsequently, this water can be stored in vessels < 20 μm (**Figure 9**). Based on these findings, we identified which pores shrub roots primarily extract water from, and which vessels effectively store the water during axial transport. The mechanism shows that soil pore structure can be the reliable indicator for shrub water uptake and storage.

Ongoing climate change is anticipated to amplify woody plant encroachment, with 13% more shrub cover worldwide by the end of this century (Caracciolo et al., 2016; Ding and Eldridge, 2024). Woody plants will have a more profound advantage in water acquisition, leading to the reduction in soil water concentrations and streamflow (Darrouzet-Nardi et al., 2006; Sadayappan et al., 2023). Meanwhile, the competitive advantages for obtaining available water of woody plants will be strengthened due to the increasing vessel size. Hence, the expansion of woody plant encroachment can intensify drought conditions. Considering the overlap of water-filled pores, organic matter, and root threshold values in the images, extracting water via CT is challenging. Previous studies have added tracers (e.g., KI and oxygen isotopes) to assist in water detection (Hu et al., 2022; Ju et al., 2024). However, tracers could impact the water uptake by roots, distorting the final observation (Keyes et al., 2017). In our study, core size reduction and elimination of impurities were performed to extract the water phase more precisely. Accuracy may be improved in future studies by the use of tracers that are less hazardous to plants, such as gold nanoparticles (Scotson et al., 2019; Ridhi et al., 2024).

## 5 Conclusion

Woody plant encroachment facilitated development of shrub xylem vessel size primarily due to increase in plant size. Specific aboveground and belowground structural units for water retention was recognized in this study. The water holding capacity of pores is highly dependent on pore size and pore shape: pores <30 μm, and pores with irregular shapes enhanced water retention. Pores >80 μm and elongated pores supported water movement. Vessel diameter, which is positively correlated with soil porosity due to root development, is a crucial indicator of vessel hydraulic function. Vessels <20 μm and >20 μm had high water retention capacity and conductivity, respectively. Irregular pores are the primary sites where plants take up water, which is subsequently retained in vessels < 20 μm. Therefore, soil pore structure can be the reliable indicator for shrub water uptake and storage. In the future, woody plant encroachment can intensify drought, and the trend may be enhanced by the development of shrub xylem vessels. Our findings revealed the water-related linkages between aboveground vegetation and belowground soil structures and may enable the prediction of hydraulic dynamics of shrubs in arid and semiarid regions in the context of global climate change.

## Data Availability

The datasets used and/or analyzed during the current study are available in the Figshare database. DOI: 10.6084/m9.figshare.28430012.
