# Supplementary material for: Water distribution and association in plant vessels and soil pores in a shrub-encroached grassland
Source: Front Plant Sci. 2025 Jul 11;16:1595608. doi: 10.3389/fpls.2025.1595608 (PMC12289628; doi:10.3389/fpls.2025.1595608)
Supplement: Supplementary file 1 [file Table1.docx]

**Supporting information**

Supplementary Table 1 Basic conditions of sampling sites

| Sampling site | Longitude | Latitude | Mean area of shrub patch (m^2^) | Shrub coverage (%) | Mean height of shrub (cm) |
| --- | --- | --- | --- | --- | --- |
| State 1 | 115°26′07″E | 42°06′35″N | 0.85 | 1.32 | 12.44 |
| State 2 | 115°27′45″E | 42°08′05″N | 3.53 | 12.96 | 13.98 |
| State 3 | 115°26′30″E | 42°06′41″N | 9.59 | 40.12 | 21.56 |

Supplementary Table 2 The 3D pore characteristics of state 1, 2 and 3

| State | Equivalent diameter (mm) | Porosity (mm^3^/mm^3^) | Surface area density (mm^2^/mm^3^) | Node density (×10^7^ no./m^3^) | Branch density (×10^8^ no./m^3^) | Mean angle (°) | Mean coordination number (no.) |
| --- | --- | --- | --- | --- | --- | --- | --- |
| State 1 | 0.142±0.003 | 0.0022±0.0006 | 0.049±0.011 | 8.74±2.50 | 1.59±0.62 | 57.54±2.27 | 3.48±0.42 |
| State 2 | 0.150±0.004 | 0.0037±0.0043 | 0.062±0.037 | 8.97±4.07 | 1.39±0.65 | 54.56±0.77 | 3.08±0.39 |
| State 3 | 0.143±0.009 | 0.0039±0.0017 | 0.060±0.034 | 9.27±5.38 | 1.74±1.08 | 55.70±2.64 | 3.82±0.61 |

Note: All data was presented with standard error (n=3). Different lowercase note represents significant differences in parameters of state 1, 2 and 3 (*p*<0.05).
